# Immunocompetent host develops mild intestinal inflammation in acute infection with *Toxoplasma gondii*

**DOI:** 10.1371/journal.pone.0190155

**Published:** 2018-01-11

**Authors:** Paulo da Silva Watanabe, Aline Rosa Trevizan, Saulo Euclides Silva-Filho, Marcelo Biondaro Góis, João Luiz Garcia, Roberto Kenji Nakamura Cuman, Ana Cristina Breithaupt-Faloppa, Débora de Mello Gonçales Sant`Ana, Gessilda de Alcantara Nogueira de Melo

**Affiliations:** 1 Biosciences and Physiopathology Program, Universidade Estadual de Maringá, Maringá, Paraná, Brazil; 2 Pharmaceutical Sciences Graduate Program, Universidade Estadual de Maringá, Maringá, Paraná, Brazil; 3 Universidade Estadual de Londrina, Londrina, Paraná, Brazil; 4 Heart Institute (InCor), LIM 11, Universidade de São Paulo—Medical School, São Paulo, São Paulo, Brazil; Universidade Estadual de Maringa, BRAZIL

## Abstract

*Toxoplasma gondii* (*T*. *gondii*) is the causative agent of toxoplasmosis, common zoonosis among vertebrates and high incidence worldwide. During the infection, the parasite needs to transpose the intestinal barrier to spread throughout the body, which may be a trigger for an inflammatory reaction. This work evaluated the inflammatory alterations of early *T*. *gondii* infection in peripheral blood cells, in the mesenteric microcirculation, and small intestinal tissue by measurement of MPO (myeloperoxidase) activity and NO (nitric oxide) level in rats. Animals were randomly assigned into control group (CG) that received saline orally and groups infected with 5,000 oocysts for 6 (G6), 12 (G12), 24 (G24), 48 (G48) and 72 hours (G72). Blood samples were collected for total and differential leukocyte count. Intravital microscopy was performed in the mesentery to evaluate rolling and adhesion of leukocytes. After euthanasia, 0.5cm of the duodenum, jejunum and ileum were collected for the determination of MPO activity, NO level and PCR to identify the parasite DNA and also the mesentery were collected to perform immunohistochemistry on frozen sections to quantify adhesion molecules ICAM-1, PECAM-1 and P-Selectin. The parasite DNA was identified in all infected groups and there was an increase in leukocytes in the peripheral blood and in expression of ICAM-1 and PECAM-1 in G6 and G12, however, the expression of P-selectin was reduced in G12. Leukocytes are in rolling process during the first 12 hours and they are adhered at 24 hours post-infection. The activity of MPO increased in the duodenum at 12 hours, and NO increased in the jejunum in G72 and ileum in G24, G48 and G72. Our study demonstrated that *T*. *gondii* initiates the infection precociously (at 6 hours) leading to a systemic activation of innate immune response resulting in mild inflammation in a less susceptible experimental model.

## Introduction

*Toxoplasma gondii* (*T*. *gondii*) is an intracellular parasite that has the ability to infect warm-blooded animals but only the felines are the definitive hosts where they can sexually reproduce. Domestic and wild animals, and humans are considered intermediate hosts, however, the parasite does not complete its life cycle [1,2].

Epidemiological studies in Brazil have shown that the prevalence of *T*. *gondii* infection in the general population ranges from 20% to 84% [3]. It is estimated that about half a billion people worldwide exhibit antibodies against this parasite [4]. Studies in Brazil, which verified seropositivity and risk factors for infection in cattle, determined that in the northern region of Brazil, only 5.3% were found [5]. However, this percentage might reach up to 71% in other regions where there is a higher production of cattle [6]. Thus being an important route of contamination.

*T*. *gondii* is transmitted primarily by ingestion of oocysts present in contaminated water and food or tissue cysts present in raw or undercooked meat [7]. During the acute infection, tachyzoites multiply rapidly and reach different host cells. Subsequently, bradyzoites form tissue cysts, preferentially in nervous and muscle tissue, characterizing the chronic phase of infection [8,9].

After oral infection, the parasite transposes the intestinal barrier and spreads through the body initiating an inflammatory response [10]. Polymorphonuclear leukocytes (PMN), the first line of defense against infectious agents, initially roll and adhere to the wall vessels of the blood microcirculation. This process is known as leukocytes rolling [11,12]. The firm adherence occurs later, by the contact of leukocytes with endothelial immunoglobulins [13]. After adherence the leukocytes transmigrate into the extravascular space towards the injury focus in attempt to combat, or at least stop the parasite [14].

Previously it has been shown that *T*. *gondii* causes intense inflammation in the intestine [15–17]. Inflammatory infiltrate was observed in the intestinal wall of domestic cats [18], birds [19] and rats [20] infected with *T*. *gondii* showing the presence of eosinophils [19]. However, there are few studies evaluating the kinetics of acute infection by *T*. *gondii* at different timepoints. Therefore, it is important to understand the parasite-host interaction that occurs during the transposition of intestinal barrier by *T*. *gondii*. This study evaluated the inflammatory changes of early *T*. *gondii* infection in peripheral blood cells, in the mesenteric microcirculation, and small intestinal tissue by measurement of MPO activity and NO level.

## Material and methods

### Experimental design

The experimental protocol was approved by the Ethics Committee on Animal Experiments of the Universidade Estadual de Maringá, Brazil (protocol number 079/2013). The experiments were performed in accordance with the guidelines of the Brazilian Control and Experimentation Committee.

Four-week-old male Wistar rats received orally antiparasitic treatment with metronidazole (500mg/kg/5 days) and fenbendazole (50mg/kg/single dose). Seven days after the end of the treatment, stool testing was performed to confirm the absence of any parasite. The rats were subsequently randomly allocated into a control group (CG) and the infected groups with *T*. *gondii* for 6 hours (G6), 12 hours (G12), 24 hours (G24), 48 hours (G48) and 72 hours (G72).

Orally each animal in the infected group received 5,000 sporulated oocysts of *T*. *gondii* (ME-49 strain, genotype II) that were resuspended in 1 mL sterile saline (S1 Fig). Oocysts were obtained from the Parasitology Veterinary Laboratory, at the Universidade Estadual de Londrina, Brazil. CG received only saline. These animals were kept under standard conditions in controlled temperature (24 ± 2°C) and photoperiod of 12 hours (6 a.m.–18 p.m.), receiving standard rodent chow (Nuvilab, Colombo, PR, BR) and water *ad libitum*.

### White blood cell counts

Immediately before euthanasia, blood samples were obtained from the tail and total leukocytes were counted using a Neubauer chamber. The differential leukocyte count was determined in blood smears stained by May-Grunwald-Giemsa using light microscopy. The data obtained were expressed as mean ± S.E.M. The percentage values were calculated by the ratio of the mean number of leukocytes in the group divided by the mean of the control group times 100.

### Euthanasia and tissue collection

After the experimental period, the rats were euthanized by deep anesthesia with halothane vapor [21]. Then, vertical laparotomy was performed and the duodenum, jejunum and ileum were excised.

### Detection of the parasite in the intestine using qPCR

*T*. *gondii* DNA was detected by real-time quantitative PCR in all infected animals used in the experiments. One centimetre of the whole wall from duodenum, jejunum and ileum were collected for this assay. For DNA extraction and precipitation by sodium acetate and ethanol was performed as described previously [22]. The amplification of *T*. *gondii* DNA was held using the method described by Homan et al. (2000). Primers Tox4 (CGCTGCAGGGAGGAAGACGAAAGTTG) and Tox5 (CGCTGCAGACACAGTGCAT CTGGATT) were used and these flanked a 529 bp fragment (GenBank No. AFI46527) of *T*. *gondii* DNA. The DNA was quantified using Quant-iT™ dsDNA Assay Kit, broad range, (Life technology).

### Intravital microscopy analysis in the mesenteric microcirculation

The procedures were adapted from Baez (1973)[23]. The rats (140–220g; n = 4–10) were anesthetized with a combination of 10 mg.kg^-1^ xylasine (Calmium Agener-Union Animal Health) and 50 mg.kg^-1^ Ketamine (Francotar®—Virbac Animal health) by intramuscular route. After trichotomy a midline abdominal incision was made and a loop of the ileal mesentery was exteriorized and their mesenteric was exposed for observation of the microcirculation vessels *in situ* by microscopy. The animals were maintained on a heated plate (37°C) provided with a transparent area over which the mesentery was fixed. The preparation was kept moist and warm by irrigation with Ringer-Locke solution (37°C, pH 7.2–7.4). Post-capillary venules with diameters ranging from 18–25 μm were analyzed (S2 Fig).

The interaction of circulating leukocytes with the surface of the endothelium was determined by counting of the number of rolling and adherent leukocytes in a segment of 100μm vessel for 5 minutes. Leukocytes that were stationary for at least 30s were considered adherent to venular endothelium. Two numerical determinations were made per animal and each vascular segment was examined only once. After the end of each experiment, euthanasia was performed by deep anesthesia. Data were expressed by the number of rolling and adherent cells/100 μm of venule over a period of 5 minutes.

### Immunohistochemical analyses of adhesion molecules in mesentery

For immunohistochemistry, only the groups CG, G6, G12 and G24 were included. After euthanasia, the mesentery was immediately removed and frozen in nitrogen-hexane solution. Cryosections (10 μm) were obtained and fixed in cold acetone for 10 min. Nonspecific sites were then blocked by incubation at room temperature with Tris-buffered saline/Tween_20_ containing bovine serum albumin (BSA) (0.5%) for 15 min. The sections were incubated for 2 h at 37°C with one of the following antibodies a mouse monoclonal anti-P-selectin antibody (CD62P; Abcam, USA), mouse monoclonal anti-ICAM-1 antibody (CD54; Santa Cruz Biotech., USA) and finally a mouse monoclonal anti-PECAM-1 antibody (CD31; Novus Biologicals, USA). The antibodies were diluted at a concentration of 1:100 in TBST (Tris-buffered Saline) containing 1% BSA. Endogenous peroxidase was blocked using 2% hydrogen peroxide (30 w/w) for 15 min at room temperature. After washing the slides with TBST, the sections were incubated with secondary antibodies (ADVANCE ™ HRP Link, Dako [PECAM-1] or Histostain ^®^ − Plus Invitrogen ^TM^ [P-selectin and ICAM-1]) for 2 h at 37°C, rinsed in TBST, and then incubated with HRP-conjugated antibodies for 30 min at room temperature. After tree washes, the samples were developed with the HRP substrate 3-amino-9-ethylcarbazole (AEC; Vector Lab., Burlingame, CA, USA) or 3, 3'-Diaminobenzidine (DAB; Invitrogen ^TM^) for 10 min and counterstained with Mayer's hematoxylin. The background reaction was determined in sections incubated in the absence of primary antibody (negative control). The slides were mounted, and images were captured using an Olympus BX50 microscope equipped with a 3CCD Pro-series camera. The objects stained with AEC and DAB on the walls of mesenteric vessels were identified after threshold determination, and the stained area was quantified using an image analyzer (NIS-elements; Nikon, Tokyo, Japan [24].

### Determination of myeloperoxidase activity on intestinal tissues

Myeloperoxidase (MPO) activity was determined using homogenate supernatant of 0.5 cm from the duodenum, jejunum and ileum. The samples were placed in 50 mM potassium phosphate buffer solution (pH 6.0) that contained 0.5% hexadecyl trimethyl ammonium bromide (1 mL/50 mg of tissue; Sigma^®^) in a potter homogenizer. The homogenate was vortexed and centrifuged for 5 min. 10 μl of the supernatant was added to each well of a 96-well microplate in duplicate. 200μl of the substrate solution (16.7mg o-dianisidine dihydrochloride (Sigma^®^), 90 mL double-distilled water, 10 mL potassium phosphate buffer, and 50 μl of 1% H_2_O_2_) were added. The enzyme reaction was stopped by the addition of 30 μl of sodium acetate. The optical density was measured at 460 nm using a microplate spectrophotometer (Asys Expert Plus).

### Measurement of total nitrite on intestinal tissues

The level of nitric oxide was determined by Griess method, which determines the nitrite production as a measure of the gas production [25]. The supernatant from the duodenum, jejunum and ileum homogenate (50 μl) was placed in a 96-well microplate in triplicate, and then, Griess solution was added (1% sulfanilamide in 5% phosphoric acid and 0.1% dihydrochloride N-1-naftiletilonodiamine in water) at room temperature. After 10 min, a reading was performed using an ELISA plate reader at a wavelength of 550 nm [26]. NO concentrations were calculated from a sodium nitrite standard curve. The results were expressed as μM.Statistical analysis

The data were expressed as mean ± SEM for each experimental group. The results were statistically evaluated using one-way analysis of variance (ANOVA) followed by Tukey’s test. The software used was GraphPad Prism version 5.01, GrahPad Software, Inc. Differences were considered significant at *p* < 0.05.

## Results

### *T*. *gondii* DNA was detectable in infected rats

The rats did not present clinical manifestations during the experiment. We performed real-time PCR to analyze the presence of the parasite DNA in the duodenum, jejunum and ileum. The results demonstrated that the T. gondii DNA was present in all infected groups, except in the ileum of G12 (Table 1), however the parasite DNA was observed later on the G24.

**Table 1:** Real-time quantitative PCR assay in the small intestine of *T*. *gondii* infected rats.

Groups	Animals	Duodenum		Jejunum		Ileum	
		Ct	fg/μL	Ct	fg/μL	Ct	fg/μL
GC	1	-	-	-	-	-	-
	2	-	-	-	-	-	-
	3	-	-	-	-	-	-
	4	-	-	-	-	-	-
G6	1	36.2	71.0	-	-	37.8	31.0
	2	29.9	1093.0	37.7	3.2	35.7	14.0
	3	35.5	105.0	-	-	34.5	20.0
	4	34.9	17.0	34.6	19.0	28.0	22570.0
G12	1	-	-	33.6	6.0	-	-
	2	-	-	33.8	40.0	-	-
	3	-	-	-	-	-	-
	4	34.7	22.0	36.6	6.0	-	-
G24	1	37.5	189.0	34.0	247.0	-	-
	2	35.4	12.0	34.7	19.0	34.7	19.0
	3	31.3	954.0	37.1	44.0	-	-
	4	34.2	26.0	33.9	31.0	35.1	18.0
G48	1	-	-	-	-	-	-
	2	35.1	15.0	36.3	8.0	-	-
	3	-	-	-	-	-	-
	4	35.7	10.0	-	-	35.8	9.0
G72	1	36.9	5.0	35.3	14.0	34.2	-
	2	34.5	210.2	34.8	18.0	35.0	26.1
	3	34.3	24.0	34.9	18.0	34.5	17.0
	4	35.0	18.0	33.8	32.0	-	21.0

Real-time PCR assay performed by using the primers Tox4 and 5 on 1 cm piece of duodenum, jejunum and ileum of control group (CG) and rats infected with *Toxoplasma gondii* (ME-49) for 6 (G6), 12 (G12), 24 (G24), 48 (G48) and 72 hours (G72). Ct, threshold cycle; (-)—not detected.

### *T*. *gondii* infection induces recruitment of predominantly PMN cells through ICAM-1 and PCAM-1

**Fig 1:**
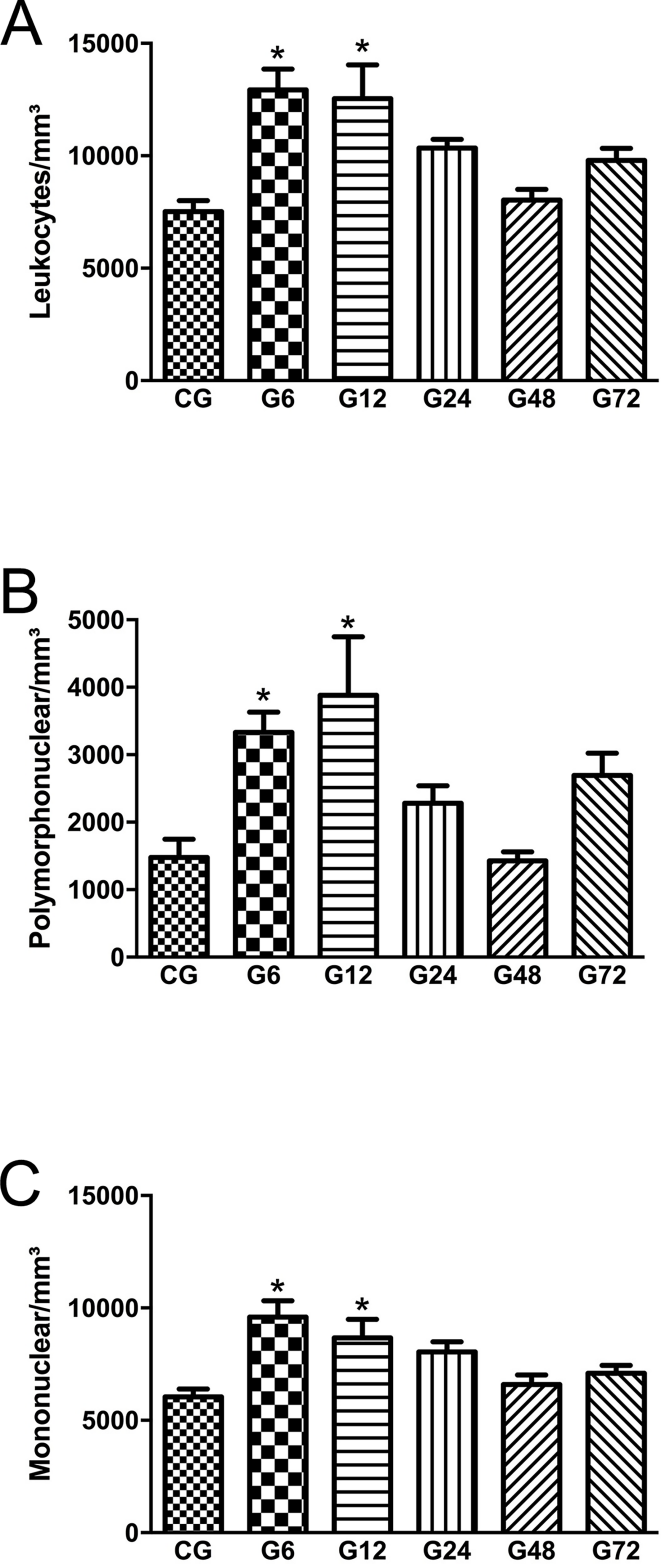
Total leucocyte count (A), Polymorphonuclear (B), and Mononuclear (C) leukocytes count in the peripheral blood of Wistar rats infected with oocysts of *T*. *gondii* at different times. Data are mean ± S.E.M., * *p* < 0.05, compared to the control group (ANOVA, Tukey’s test). CG (Control group), G6, G12, G24 G48 and G72 (rats infected for 6, 12, 24, 48 or 72 hours with oocysts of ME 49 strain of *T*. *gondii*, respectively).

We evaluated the mobilization of leukocytes in the systemic circulation towards to the tissue. Firstly, we counted the number of total leukocytes in the peripheral blood which were increased only between 6h and 12h. Total leukocytes were bigger in 72% at 6h (G6: 12,928 ± 933 mm^-3^) and in 69% at 12h post infection (G12: 12,544 ± 1,495 mm^-3^) when compared to CG (7,516 ± 494 mm^-3^) (*p* < 0.05). Additionally, when we investigated the differential leukocyte counting, a massive increase of PMN rather than MN was observed. PMN were 125% bigger at 6h (G6: 3,331 ± 302 mm^-3^) and 163% at 12h post infection (G12: 3,880 ± 869 mm^-3^) in relation to CG, while MN were 59% at 6h (G6: 9,597 ± 723 mm^-3^) and 43% at 12h post infection (G12: 8,665 ± 829 mm^-3^) (*p* < 0.05) (Fig 1) also in relation to CG. These results showed that the PMN cell type are mostly recruited in early infection of this experimental model. Furthermore, the number of these cells was normalized after 24h post infection (S1 Table.).

**Fig 2:**
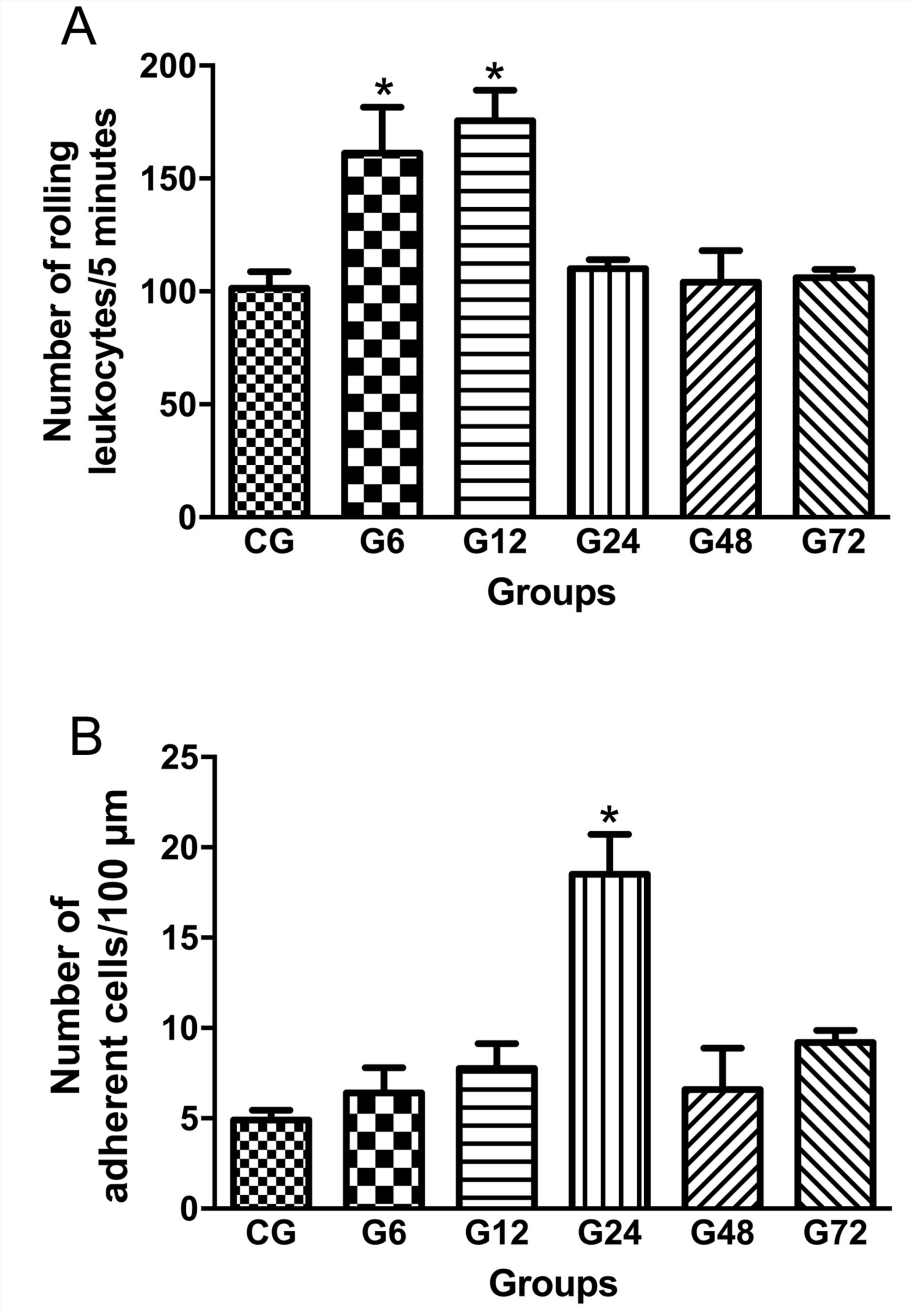
Number of rolling (A) and adherent leukocytes/100 μm venule length (B) in mesenteric microcirculation from distal ileum region of rats orally infected with *T*. *gondii* oocysts (ME49). Data are mean ± S.E.M., * *p* < 0.05, compared to the control group (ANOVA, Tukey’s test). CG (Control group), G6, G12, G24 G48 and G72 (rats infected for 6, 12, 24, 48 or 72 hours with oocysts of ME 49 strain of *T*. *gondii*, respectively).

**Fig 3:**
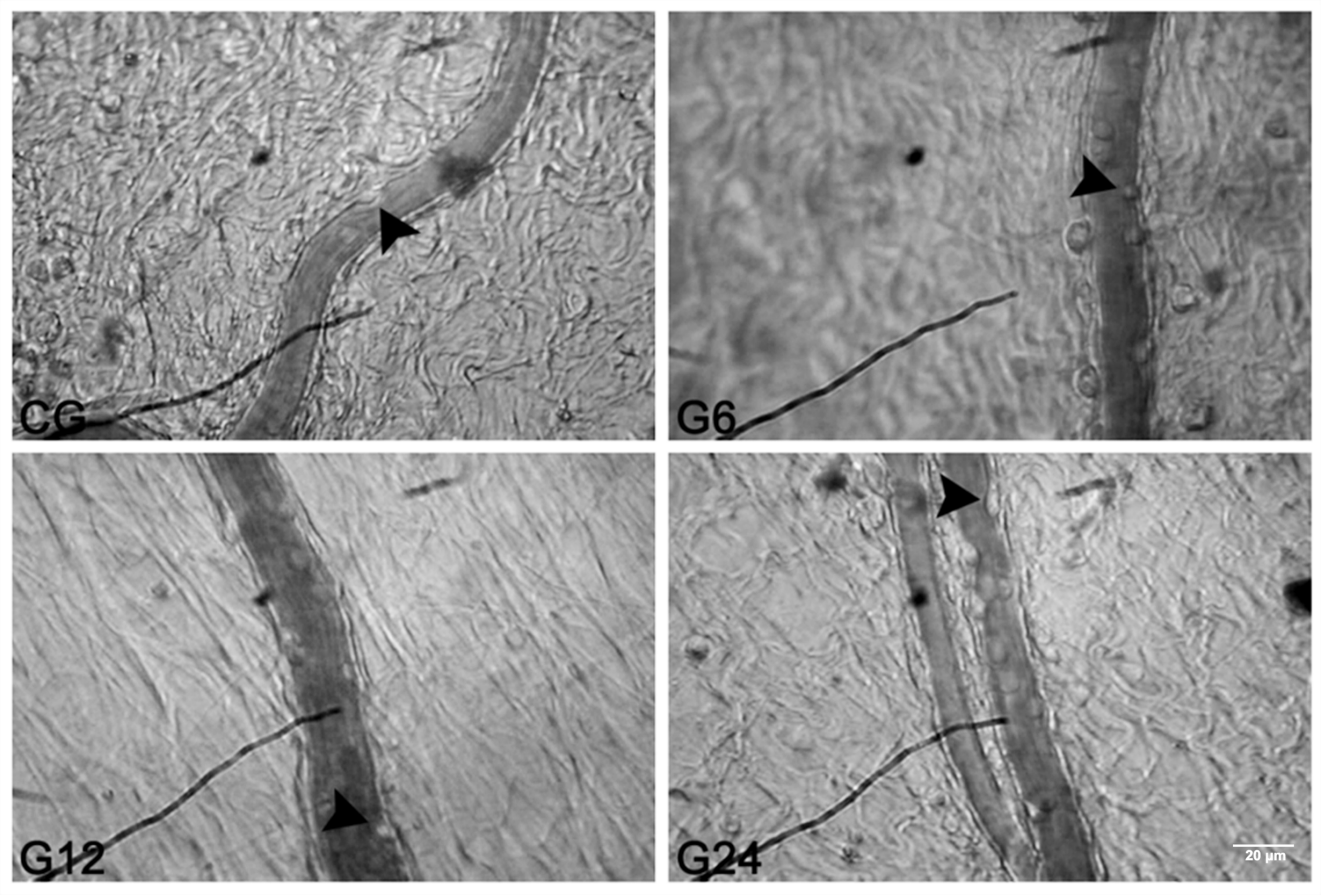
Representative photomicrographs of rat mesenteric microcirculation. CG (Control group), G6, G12 and G24 (infected for 6, 12 and 24 hours with oocysts of ME 49 strain of *T*. *gondii*). Increased number of rolling leukocytes was observed in the G6 and G12 and number of adherent in G24 in comparison with CG. Leukocytes (arrow). Final magnification: 3400x.

In order to evaluate the recruitment of leukocytes from the peripheral blood towards to intestinal tissue, we performed the mesenteric microcirculation *in situ*. The *T*. *gondii* infection caused a marked increase in the rolling leukocytes number from 6h (G6: 161.2 ± 20.4 leukocytes.5 min^-1^) up to 12h (G12: 175.6 ± 13.5 leukocytes.5 min^-1^) post infection (*p* < 0.05) when compared to the CG (101.0 ± 7.4 cells/5 min) (Fig 2A). Whilst, no significant differences was observed for rolling leukocytes in the G24 (110.1 ± 3.9 cells/5 min), G48 (104.0 ± 14.1 cells/5 min) or G72 (106.0 ± 3.7 cells/5 min) (*p*>0.05). Furthermore, the infection resulted in an increase of adherent cells only in the G24 (18.5 ± 2.2 cells/100 μm) *(p* < 0.05) when compared to CG (5.0 ± 2.0 cells/100 μm). The number of adherent cells remained unchanged in the G6 (6.4 ± 1.42 cells/100 μm), G12 (7.7 ± 1,4 cells/100 μm), G48 (6.6 ± 2.3 cells/100 μm) and G72 (9.2 ± 0.7 cells/100 μm) in relation to CG (Fig 2B; *p*>0.05). These numerical changes are illustrated in the Fig 3.

**Fig 4:**
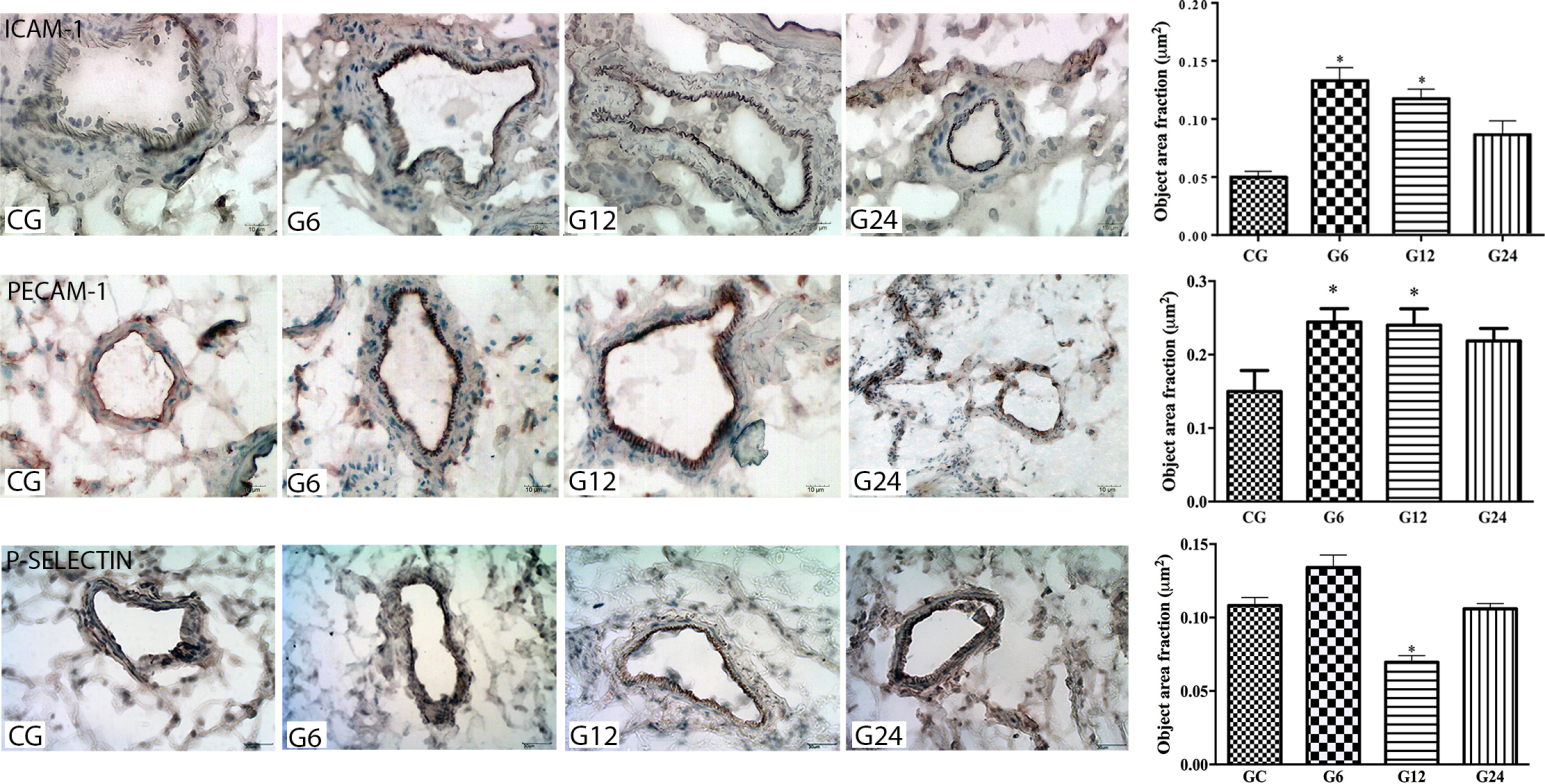
Representative photomicrograph and graphs for immunohistochemical evaluation of the expression of ICAM-1, PECAM-1 and P-selectin molecules on the mesenteric vascular endothelium of rats orally infected with *T*. *gondii* oocysts (ME49). Data are mean ± S.E.M. * *p* < 0.05, compared to the control group (ANOVA, Tukey’s test). CG (Control group), G6, G12, G24 G48 and G72 (rats infected for 6, 12, 24, 48 or 72 hours with oocysts of ME 49 strain of *T*. *gondii*, respectively).

We evaluated the adhesion molecules in the mesenteric endothelium involved in the migration of leukocytes (Fig 4) and we observed that the expression of ICAM-1 and PECAM-1 were statistically augmented in the G6 and G12 groups compared to control (*p* < 0.05). It means that ICAM-1 and PECAM-1 were the main adhesion molecules involved in the migration process of leukocytes during the *T*. *gondii* infection. On the other hand, we observed that P-selectin was decreased only in the G12 (*p* < 0.05).

### *T*. *gondii* infection impaired MPO activity and increased the release of NO

After the migration of leukocytes to the gut tissue, we evaluated the activity of immune cells through MPO and release of NO. As shown below, NO demonstrated to be more relevant to combat the parasite.

**Fig 5:**
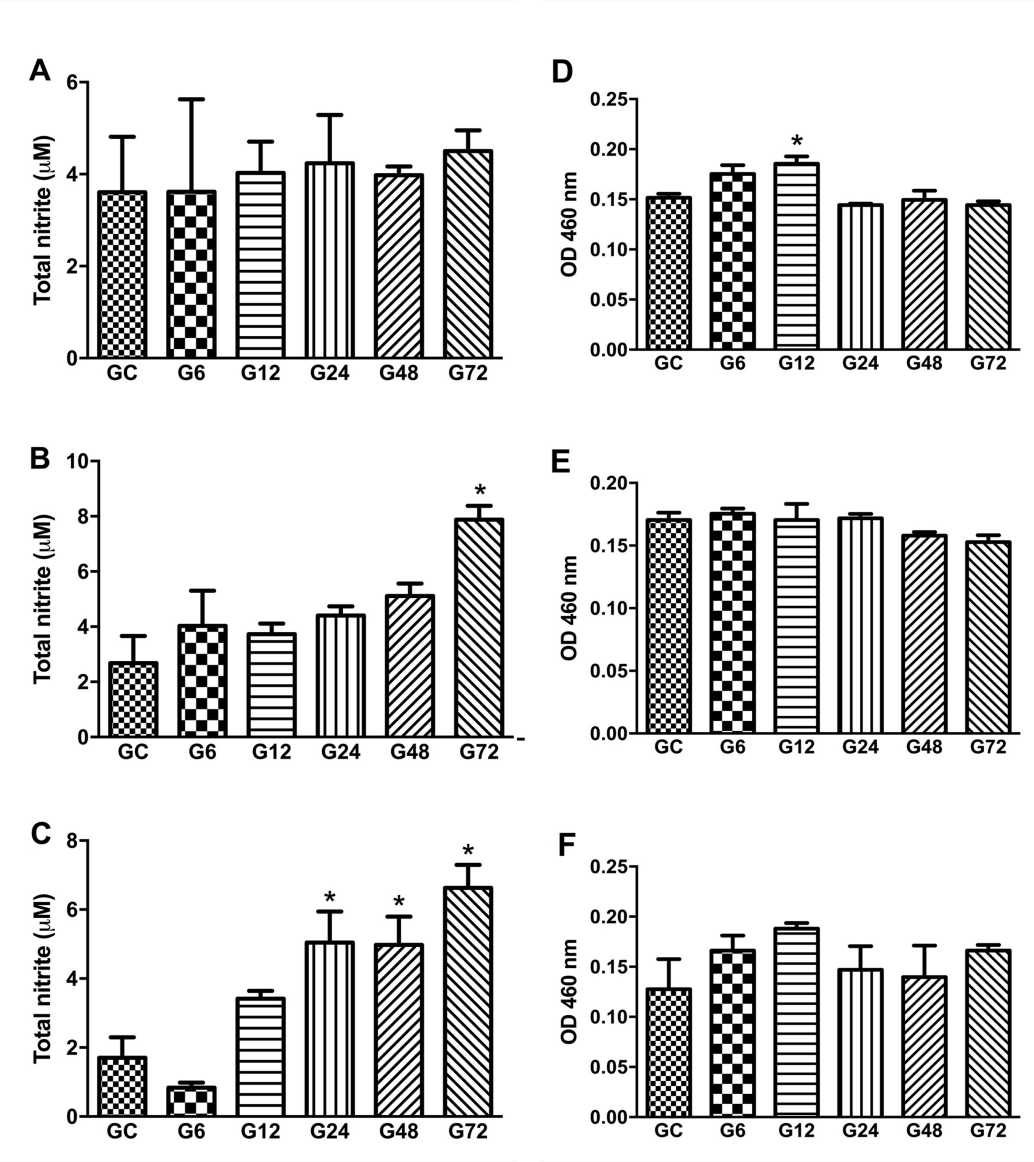
Myeloperoxidase activity in duodenum (A), jejunum (B) and ileum (C) and nitrite dosage as an indirect way to measure nitric oxide in the duodenum (D), jejunum (E) and ileum (F) at different times of early infection with oocysts of *T*. *gondii*. Data are mean ± S.E.M., * *p* < 0.05, compared to the control group (ANOVA, Tukey’s test). CG (Control group), G6, G12, G24 G48 and G72 (rats infected for 6, 12, 24, 48 or 72 hours with oocysts of ME 49 strain of *T*. *gondii*, respectively).

The level of NO production was monitored by measuring the nitrite level in the small intestine by Griess reaction in order to evaluate the release of NO from resident/migrated cells in the acute *T*. *gondii* infection. The nitrite quantification showed an increased NO production in the jejunum of G72 (7.882 ± 0.995 μM) and in ileum of the groups G24 (5.043 ± 1.804 μM), G48 (4.974 ± 1.640 μM), and G72 (6.627 ± 1.165 μM) in comparison to the CG (2.680 ± 1.709 for jejunum and 1.701 ± 1.033 μM for ileum respectively) (*p* < 0.05) (Fig 5A, 5B and 5C).

We assayed the MPO activity to confirm the presence/activation of neutrophils in the small intestine. The results showed a significant increase of MPO activity in the duodenum only at 12h post infection (G12: 0.1856 ± 0.0143) (*p*<0.05) compared to the control group (0.1516 ± 0.0078). On the other hand, the infection did not modify the MPO activity in the jejunum and ileum of the rats (*p* > 0.05) (Fig 5D, 5E and 5F). These results show that the main immune response to *T*. *gondii* is not through MPO activity.

## Discussion

Information about the innate response in immunocompetent hosts at the beginning of *T*. *gondii* infection is limited. Here we used a rat model of oral *T*. *gondii* infection to study some inflammatory alterations at the site of infection, in the small intestine and mesentery.

Our data showed an increase in leukocyte cells in peripheral blood at the beginning of infection. Furthermore, on differential leukocyte count, it is clear that this increase was mainly by PMN, showing that these cells are the most recruited during early infection (at 6 hours) in this experimental model. Studies have shown that depletion of PMN promotes an increased susceptibility of the host to infection by *T*. *gondii*, however, the removal of the PMN does not interfere in later stages of the disease [27,28]. Therefore neutrophils are very important in early infection since they might influence the type of immune response and the control of tachyzoite replication [10,28,29].

We showed using qPCR that at 6 hours after oocysts inoculation the *T*. *gondii* DNA was present throughout the small intestine and remains in all segments for at least 72 hours, even in the ileum, which is farthest from the local of *T*. *gondii* inoculation. These results associated with the increase in the number of blood leukocytes led us to consider that the infectious forms of the parasite have already initiated the infection of the intestinal epithelial cells. In this context, the defense cells are mobilized from the blood to reach the site of infection. Other authors have shown that after eight hours, the parasite is internalized into phagocytic and non-phagocytic cells of the new host [30].

We used intravital microscopy in order to evaluate the leukocytes behaviour in the mesenteric microcirculation and to study the leukocytes-endothelium interaction following the initial effects of oral *T*. *gondii* infection. Our results showed that there was an increased number of leukocytes rolling in the G6 and G12 groups and adherent cells in the G24 group. These hemodynamic changes happen at the beginning of inflammation, in which vascular endothelium enhances the expression of a series of membrane molecules to interact with circulating leukocytes, among them ICAM-1, PECAM-1 and P-selectin [31]. We also observed an increased expression of ICAM- and PECAM-1 also in G6 and G12 groups, showing the sequence of events preceding the leukocytes transmigration. This result has already been expected since the *T*. *gondii* adhesin (MIC 2) interacts with ICAM-1 to adhere and transmigrate across cellular barriers [32], which could contribute to the spread of the parasite.

Unexpectedly, we also observed that P-selectin expression on mesenteric vessels was decreased in G12, although later the values were similar to the control group. Indeed, it was demonstrated that oral *T*. *gondii* infection induced the expression of P-selectin ligands on T cells without facilitating the cell recruitment into inflamed small intestine [33]. Our results suggest that the infection could be reducing P-selectin expression, since *T*. *gondii* can modulate cytokines expression and other adhesion molecules such as those from the extracellular matrix [34–37]. While, in macrophages obtained from mice, the parasite is able to increase IL12 secretion [38–40] it can also inhibit the TNFα production [41] by disabling the NFkB activation pathway, probably to avoid excessive proinflammatory cytokines production [42].

After the rolling process, these leukocytes firmly adhere to the vessel in an attempt to migrate to the site of infection to combat the causative agent of injury [13,43]. Previous studies from our group showed an increase in the number of intraepithelial lymphocytes after 48 hours of infection in rats [44].

Despite the fact that rats are considered a resistant model of toxoplasmosis, the changes observed indicate the beginning of an inflammatory process after infection. This is different from the experimental models using C57BL/6 that the infection by *T*. *gondii* causes excessive inflammation, increasing cytokine production and a large accumulation of leukocytes in the intestine [45]. The major histopathological changes occur in the distal segments of the small intestine after 72 hours of infection in mice [46]. Our experimental model develops a mild inflammation in the small intestine and does not present clinical signals of infection.

We suggest that after six hours of contact with infective forms, these have begun to cause injury to the intestine. At the site of inflammation, neutrophils try to combat the parasite through various pathways, including its enzymes. The MPO enzyme of neutrophils and other cells of myeloid origin is responsible for catalyzing the production of hypochlorous acid that kill the parasites. Studies have assigned the dosage of the MPO enzyme as an indirect indicator of neutrophil presence/activation in tissues [47,48]. In our study, the MPO activity increased only in the duodenum at 12 h of infection. The parasite possibly remains viable whereas microbicidal mechanisms of neutrophils are not activated as indicated by the non-increase of MPO activity found in this study. Hence, leukocytes that enter the small intestine are not able to eradicate the parasite.

*T*. *gondii* also has the ability to alter gene expression of the host cell, and may even avoid apoptosis by preventing the activation of the mitochondrial pathway of caspase [49] facilitating their replication within the cell [50]. Neutrophils are a reservoir for *T*. *gondii*, and it is believed that this fact helps the parasite to spread through the organism using their migration capability to reach other locations [10].

The nitric oxide synthesis by macrophages is an important mechanism microbicide for a variety of intracellular pathogens, including *T*. *gondii* [51] and possible neuroprotective action. There is an increased NO production by endothelial cells in the brain of mice after infection with ME-49 strain of *T*. *gondii* [52]. The findings about NO are consistent with later recruitment of mononuclear cells as observed by others [39]. We observed that the infected ileum responds more than the duodenum or jejunum probably due to the increased number of Peyer's Patches in this segment, which is part of mucosal-associated lymphoid tissue (MALT) containing T and B lymphocytes, dendritic cells and macrophages [53]. A previous study using mice showed that after infection there is an increase in CD4^+^ T cells, interferon-γ, tumor necrosis factor-α and inducible nitric oxide synthetase that mediate the development of ileus necrosis and subsequently lead to the death of the mice in 13 days [45]. However, rats might represent a compatible model of immunocompetent host, such as *T*. *gondii* infected immunocompetent humans. In this context, we questioned whether humans seropositive for *T*. *gondii* are more susceptible to inflammation in the intestine.

Our study was the first to demonstrate that the parasite parallel to the transposition of the intestinal barrier initiate the infection precociously (at 6 hours) leading to a systemic activation of innate immune response resulting in mild inflammation in a less susceptible experimental model of *T*. *gondii* infection.

## Conclusion

Our results demonstrated that the response to *T*. *gondii* infection begins early even in immunocompetent hosts who do not develop clinical signs. At the same time, a sequence of systemic alterations detected by leukocyte mobilization in the peripheral blood and mesenteric microcirculation leads to a mild inflammatory response. Therefore, the parasite spreads through the body and resides in the intestine. It is important to conduct studies in an immunocompetent experimental model because the great majority of the human population seropositive to *T*. *gondii* antigens is also asymptomatic, but might trigger an inflammatory response similar to that seen in inflammatory bowel diseases.

## Supporting information

S1 Fig Experimental design. Schematic model exemplifying the experimental model used.
(PDF)

S2 Fig Intravital microscopy analysis. Schematic illustrating the step-wise protocol of the mesenteric microcirculation *in situ* in order to analyse the leukocyte endothelium interaction.
(PDF)

S1 Table Total and differential leukocyte count in rat peripheral blood. Total and differential leukocyte count (Cells mm-3) in rat peripheral blood. The rats of the infected groups received orally 5,000 sporulated oocysts of *T*. *gondii* (ME-49 strain, genotype II) that were resuspended in 1 mL sterile saline for 6 hours (G6), 12 hours (G12), 24 hours (G24), 48 hours (G48) and 72 hours (G72). The control group (CG) has received 1 mL sterile saline.
(PDF)

## References

[1] Dubey JP. Advances in the life cycle of *Toxoplasma gondii*. Int J Parasitol. 1998;28: 1019–1024. doi: 10.1016/S0020-7519(98)00023-X 9724872 10.1016/s0020-7519(98)00023-x

[2] Dubey JP. History of the discovery of the life cycle of *Toxoplasma gondii*. Int J Parasitol. Australian Society for Parasitology Inc.; 2009;39: 877–82. doi: 10.1016/j.ijpara.2009.01.005 19630138 10.1016/j.ijpara.2009.01.005

[3] Dubey JP, Lago EG, Gennari SM, Su C, Jones JL. Toxoplasmosis in humans and animals in Brazil: high prevalence, high burden of disease, and epidemiology. Parasitology. 2012;139: 1375–1424. doi: 10.1017/S0031182012000765 22776427 10.1017/S0031182012000765

[4] Leite M, Siciliano S, Rocha LS a, Justa MTR, César KR, Granato CFH. Correlation between specific IgM levels and percentage IgG-class antibody avidity to *Toxoplasma gondii*. Rev Inst Med Trop Sao Paulo. 2008;50: 237–42. doi: 10.1590/S0036-46652008000400010 18813765 10.1590/s0036-46652008000400010

[5] de Souza JBR, Soares VE, Maia MO, Pereira CM, Ferraudo AS, Cruz BC, et al Spatial distribution and risk factors for *Toxoplasma gondii* seropositivity in cattle slaughtered for human consumption in Rondônia, North region, Brazil. Vet Parasitol. 2016;226: 145–149. doi: 10.1016/j.vetpar.2016.07.015 27514900 10.1016/j.vetpar.2016.07.015

[6] Santos TR, Costa AJ, Toniollo GH, Luvizotto MCR, Benetti AH, Santos RR, et al Prevalence of anti-*Toxoplasma gondii* antibodies in dairy cattle, dogs, and humans from the Jauru micro-region, Mato Grosso state, Brazil. Vet Parasitol. 2009;161: 324–326. doi: 10.1016/j.vetpar.2009.01.017 19232473 10.1016/j.vetpar.2009.01.017

[7] Speer CA, Dubey JP. Ultrastructure of early stages of infections in mice fed *Toxoplasma gondii* oocysts. Parasitology. 1998;116 (Pt 1: 35–42. Available: http://www.ncbi.nlm.nih.gov/pubmed/9481772 9481772 10.1017/s0031182097001959

[8] Dubey JP. Refinement of pepsin digestion method for isolation of *Toxoplasma gondii* from infected tissues. Vet Parasitol. 1998;74: 75–7. doi: 10.1016/S0304-4017(97)00135-0 9493311 10.1016/s0304-4017(97)00135-0

[9] Hill D, Dubey JP. *Toxoplasma gondii*: Transmission, diagnosis, and prevention. Clin Microbiol Infect. 2002;8: 634–640. doi: 10.1046/j.1469-0691.2002.00485.x 12390281 10.1046/j.1469-0691.2002.00485.x

[10] Coombes JL, Charsar B a, Han S-J, Halkias J, Chan SW, Koshy A a, et al Motile invaded neutrophils in the small intestine of *Toxoplasma gondii*-infected mice reveal a potential mechanism for parasite spread. Proc Natl Acad Sci U S A. 2013;110: E1913–22. doi: 10.1073/pnas.1220272110 23650399 10.1073/pnas.1220272110 PMC3666704

[11] Turner MD, Nedjai B, Hurst T, Pennington DJ. Cytokines and chemokines: At the crossroads of cell signalling and inflammatory disease. Biochim Biophys Acta—Mol Cell Res. Elsevier B.V.; 2014;1843: 2563–2582. doi: 10.1016/j.bbamcr.2014.05.014 24892271 10.1016/j.bbamcr.2014.05.014

[12] Hawkins PT, Stephens LR. PI3K signalling in inflammation. Biochim Biophys Acta—Mol Cell Biol Lipids. 2015;1851: 882–897. doi: 10.1016/j.bbalip.2014.12.006 25514767 10.1016/j.bbalip.2014.12.006

[13] Woodfin A, Voisin M-B, Nourshargh S. Recent developments and complexities in neutrophil transmigration. Curr Opin Hematol. 2010;17: 9–17. doi: 10.1097/MOH.0b013e3283333930 19864945 10.1097/MOH.0b013e3283333930 PMC2882030

[14] Wilson DC, Grotenbreg GM, Liu K, Zhao Y, Frickel EM, Gubbels MJ, et al Differential regulation of effector- and central-memory responses to *Toxoplasma gondii* infection by IL-12 revealed by tracking of Tgd057-specific CD8+ T cells. PLoS Pathog. 2010;6 doi: 10.1371/journal.ppat.1000815 20333242 10.1371/journal.ppat.1000815 PMC2841619

[15] Liesenfeld O, Kosek J, Remington JS, Suzuki Y. Association of CD4+ T cell-dependent, interferon-gamma-mediated necrosis of the small intestine with genetic susceptibility of mice to peroral infection with *Toxoplasma gondii*. J Exp Med. 1996;184: 597–607. Available: http://www.ncbi.nlm.nih.gov/pubmed/8760813 8760813 10.1084/jem.184.2.597 PMC2192709

[16] Miller CM, Boulter NR, Ikin RJ, Smith NC. The immunobiology of the innate response to *Toxoplasma gondii*. Int J Parasitol. Australian Society for Parasitology Inc.; 2009;39: 23–39. doi: 10.1016/j.ijpara.2008.08.002 18775432 10.1016/j.ijpara.2008.08.002

[17] Gregg B, Taylor BC, John B, Tait-Wojno ED, Girgis NM, Miller N, et al Replication and distribution of *toxoplasma gondii* in the small intestine after oral infection with tissue cysts. Infect Immun. 2013;81: 1635–1643. doi: 10.1128/IAI.01126-12 23460516 10.1128/IAI.01126-12 PMC3647985

[18] Silva JM da, Silva AV da, Araújo EJ de A, Sant’ana D de MG. Efeitos da infecÇão crônica por *Toxoplasma gondii* sobre a parede intestinal de gatos domésticos. Rev Bras Parasitol Veterinária. 2010;19: 57–63. 10.4322/rbpv.01901010 20385061

[19] Bonapaz R dos S, Hermes-Uliana C, Santos F do N, Silva AV da, Araújo EJ de A, Sant’Ana D de MG. Effects of infection with *Toxoplasma gondii* oocysts on the intestinal wall and the myenteric plexus of chicken (Gallus gallus). Pesqui Veterinária Bras. 2010;30: 787–792. doi: 10.1590/S0100-736X2010000900013

[20] Rachinel N, Buzoni-Gatel D, Dutta C, Mennechet FJD, Luangsay S, Minns L a, et al The Induction of Acute Ileitis by a Single Microbial Antigen of *Toxoplasma gondii*. J Immunol. 2004;173: 2725–2735. doi: 10.4049/jimmunol.173.4.2725 15294991 10.4049/jimmunol.173.4.2725

[21] Vivas LA de M, Jamel N, Refinetti RA, Silva LF da, Rodrigues LV, Silva PC, et al Anesthetic experimental device for small animal. Acta Cir Bras. 2007;22: 229–233. doi: 10.1590/S0102-86502007000300012 17546297 10.1590/s0102-86502007000300012

[22] Homan WL, Vercammen M, De Braekeleer J, Verschueren H. Identification of a 200- to 300-fold repetitive 529 bp DNA fragment in *Toxoplasma gondii*, and its use for diagnostic and quantitative PCR. Int J Parasitol. 2000;30: 69–75. doi: 10.1016/S0020-7519(99)00170-8 10675747 10.1016/s0020-7519(99)00170-8

[23] Baez S. An open cremaster muscle preparation for the study of blood vessels by in vivo microscopy. Microvasc Res. 1973;5: 384–394. doi: 10.1016/0026-2862(73)90054-X 4709735 10.1016/0026-2862(73)90054-x

[24] Guarda IFMS, Correia CJ, Breithaupt-Faloppa AC, Ferreira SG, Moreno ACR, Martinez MB, et al Effects of ethyl pyruvate on leukocyte-endothelial interactions in the mesenteric microcirculation during early sepsis treatment. Clinics (Sao Paulo). Hospital das Clinicas da Faculdade de Medicina da Universidade de Sao Paulo; 2015;70: 508–14. doi: 10.6061/clinics/2015(07)08 26222821 10.6061/clinics/2015(07)08 PMC4496755

[25] Green LC, Wagner D a, Glogowski J, Skipper PL, Wishnok JS, Tannenbaum SR. Analysis of nitrate, nitrite, and [15N]nitrate in biological fluids. Anal Biochem. 1982;126: 131–8. doi: 10.1016/0003-2697(82)90118-X 7181105 10.1016/0003-2697(82)90118-x

[26] Saleh TS, Calixto JB, Medeiros YS. Effects of anti-inflammatory drugs upon nitrate and myeloperoxidase levels in the mouse pleurisy induced by carrageenan. Peptides. 1999;20: 949–56. doi: 10.1016/S0196-9781(99)00086-8 10503773 10.1016/s0196-9781(99)00086-8

[27] Bliss SK, Zhang Y, Denkers EY. Murine neutrophil stimulation by *Toxoplasma gondii* antigen drives high level production of IFN-gamma-independent IL-12. J Immunol. 1999;163: 2081–8. Available: http://www.ncbi.nlm.nih.gov/entrez/query.fcgi?cmd=Retrieve&db=PubMed&dopt=Citation&list_uids=10438947 10438947

[28] Bliss SK, Gavrilescu LC, Alcaraz a., Denkers EY. Neutrophil depletion during *Toxoplasma gondii* infection leads to impaired immunity and lethal systemic pathology. Infect Immun. 2001;69: 4898–4905. doi: 10.1128/IAI.69.8.4898-4905.2001 11447166 10.1128/IAI.69.8.4898-4905.2001 PMC98580

[29] Denkers EY, Butcher B a., Del Rio L, Bennouna S. Neutrophils, dendritic cells and Toxoplasma. Int J Parasitol. 2004;34: 411–421. doi: 10.1016/j.ijpara.2003.11.001 15003500 10.1016/j.ijpara.2003.11.001

[30] Hill DE, Chirukandoth S, Dubey JP. Biology and epidemiology of *Toxoplasma gondii* in man and animals. Anim Heal Res Rev. 2005;6: 41–61. doi: 10.1079/AHR2005100 10.1079/ahr2005100 16164008

[31] Park S, Sorenson CM, Sheibani N. PECAM-1 isoforms, eNOS and endoglin axis in regulation of angiogenesis. Clin Sci (Lond). 2015;129: 217–34. doi: 10.1042/CS20140714 25976664 10.1042/CS20140714 PMC4716661

[32] Barragan A, Brossier F, Sibley LD. Transepithelial migration of *Toxoplasma gondii* involves an interaction of intercellular adhesion molecule 1 (ICAM-1) with the parasite adhesin MIC2. Cell Microbiol. 2005;7: 561–8. doi: 10.1111/j.1462-5822.2005.00486.x 15760456 10.1111/j.1462-5822.2005.00486.x

[33] Hoffmann U, Pink M, Lauer U, Heimesaat MM, Winsauer C, Kruglov A, et al Regulation and migratory role of P-selectin ligands during intestinal inflammation. Waisman A, editor. PLoS One. Public Library of Science; 2013;8: e62055 doi: 10.1371/journal.pone.0062055 23630623 10.1371/journal.pone.0062055 PMC3632518

[34] Niehus S, Elass E, Coddeville B, Guérardel Y, Schwarz RT, Debierre-Grockiego F. Glycosylphosphatidylinositols of *Toxoplasma gondii* induce matrix metalloproteinase-9 production and degradation of galectin-3. Immunobiology. 2012;217: 61–4. doi: 10.1016/j.imbio.2011.08.001 21924517 10.1016/j.imbio.2011.08.001

[35] Cordeiro CA, Vieira ELM, Castro VM, Dutra WO, Costa RA, Orefice JL, et al T cell Immunoregulation in Active Ocular Toxoplasmosis. Immunol Lett. Elsevier B.V.; 2017; doi: 10.1016/j.imlet.2017.02.009 28214536 10.1016/j.imlet.2017.02.009

[36] Coutermarsh-Ott SL, Doran JT, Campbell C, Williams TM, Lindsay DS, Allen IC. Caspase-11 Modulates Inflammation and Attenuates *Toxoplasma gondii* Pathogenesis. Mediators Inflamm. Hindawi Publishing Corporation; 2016;2016: 9848263 doi: 10.1155/2016/9848263 27378827 10.1155/2016/9848263 PMC4917705

[37] Unno A, Kitoh K, Takashima Y. Up-regulation of hyaluronan receptors in *Toxoplasma gondii*-infected monocytic cells. Biochem Biophys Res Commun. Elsevier Inc.; 2010;391: 477–80. doi: 10.1016/j.bbrc.2009.11.083 19914206 10.1016/j.bbrc.2009.11.083

[38] Butcher B a, Denkers EY. Mechanism of entry determines the ability of *Toxoplasma gondii* to inhibit macrophage proinflammatory cytokine production. Infect Immun. 2002;70: 5216–24. doi: 10.1128/IAI.70.9.5216-5224.2002 12183573 10.1128/IAI.70.9.5216-5224.2002 PMC128277

[39] Cohen SB, Maurer KJ, Egan CE, Oghumu S, Satoskar AR, Denkers EY. CXCR3-Dependent CD4+ T Cells Are Required to Activate Inflammatory Monocytes for Defense against Intestinal Infection. Sibley LD, editor. PLoS Pathog. 2013;9: e1003706 doi: 10.1371/journal.ppat.1003706 24130498 10.1371/journal.ppat.1003706 PMC3795032

[40] Almeida F, Sardinha-Silva A, Da Silva TA, Pessoni AM, Pinzan CF, Alegre-Maller ACP, et al *Toxoplasma gondii* chitinase induces macrophage activation. PLoS One. 2015;10: 1–12. doi: 10.1371/journal.pone.0144507 26659253 10.1371/journal.pone.0144507 PMC4684212

[41] Butcher B a, Denkers EY. Mechanism of Entry Determines the Ability of *Toxoplasma gondii* To Inhibit Macrophage Proinflammatory Cytokine Production Mechanism of Entry Determines the Ability of *Toxoplasma gondii* To Inhibit Macrophage Proinflammatory Cytokine Production. 2002;70: 5216–5224. doi: 10.1128/IAI.70.9.5216-5224.2002 12183573 10.1128/IAI.70.9.5216-5224.2002 PMC128277

[42] Butcher B a, Kim L, Johnson PF, Denkers EY. *Toxoplasma gondii* tachyzoites inhibit proinflammatory cytokine induction in infected macrophages by preventing nuclear translocation of the transcription factor NF-kappa B. J Immunol. 2001;167: 2193–201. doi: 10.4049/jimmunol.167.4.2193 11490005 10.4049/jimmunol.167.4.2193

[43] Sperandio M, Smith ML, Forlow SB, Olson TS, Xia L, McEver RP, et al P-selectin glycoprotein ligand-1 mediates L-selectin-dependent leukocyte rolling in venules. J Exp Med. 2003;197: 1355–63. doi: 10.1084/jem.20021854 12756271 10.1084/jem.20021854 PMC2193782

[44] Trevizan AR, Vicentino-Vieira SL, da Silva Watanabe P, Góis MB, de Melo G de AN, Garcia JL, et al Kinetics of acute infection with *Toxoplasma gondii* and histopathological changes in the duodenum of rats. Exp Parasitol. 2016;165: 22–29. doi: 10.1016/j.exppara.2016.03.015 26993084 10.1016/j.exppara.2016.03.015

[45] Liesenfeld O. Oral infection of C57BL/6 mice with *Toxoplasma gondii*: a new model of inflammatory bowel disease? J Infect Dis. 2002;185 Suppl: S96–101. doi: 10.1086/338006 11865446 10.1086/338006

[46] Dubey JP, Ferreira LR, Martins J, McLeod R. Oral oocyst-induced mouse model of toxoplasmosis: effect of infection with *Toxoplasma gondii* strains of different genotypes, dose, and mouse strains (transgenic, out-bred, in-bred) on pathogenesis and mortality. Parasitology. 2012;139: 1–13. doi: 10.1017/S0031182011001673 22078010 10.1017/S0031182011001673 PMC3683600

[47] Nacif LS, Andraus W, Kubrusly MS, Molan N, Chaib E, D’Albuquerque LAC. Atividade da mieloperoxidase está aumentada na síndrome hepatopulmonar em ratos. ABCD Arq Bras Cir Dig (São Paulo). 2013;26: 293–295. doi: 10.1590/S0102-67202013000400008 10.1590/s0102-67202013000400008 24510037

[48] Winterbourn CC, Kettle AJ. Biomarkers of myeloperoxidase-derived hypochlorous acid. Free Radic Biol Med. 2000;29: 403–9. doi: 10.1016/S0891-5849(00)00204-5 11020661 10.1016/s0891-5849(00)00204-5

[49] Goebel S, Gross U, Lüder CG. Inhibition of host cell apoptosis by *Toxoplasma gondii* is accompanied by reduced activation of the caspase cascade and alterations of poly(ADP-ribose) polymerase expression. J Cell Sci. 2001;114: 3495–505. 11682609 10.1242/jcs.114.19.3495

[50] Dunn JD, Butcher B, Denkers E, Boothroyd J. to Toxoplasma gondii. 2007; 0–12.

[51] Khan I a, Schwartzman JD, Matsuura T, Kasper LH. A dichotomous role for nitric oxide during acute *Toxoplasma gondii* infection in mice. Proc Natl Acad Sci U S A. 1997;94: 13955–60. 9391134 10.1073/pnas.94.25.13955 PMC28414

[52] Dincel GC, Atmaca HT. Nitric oxide production increases during *Toxoplasma gondii* encephalitis in mice. Exp Parasitol. Elsevier Inc.; 2015;156: 104–12. doi: 10.1016/j.exppara.2015.06.009 26115941 10.1016/j.exppara.2015.06.009

[53] Jung C, Hugot J-P, Barreau F. Peyer’s Patches: The Immune Sensors of the Intestine. Int J Inflam. 2010;2010: 1–12. doi: 10.4061/2010/823710 21188221 10.4061/2010/823710 PMC3004000

