# Supplementary figures and images for: Immunocompetent host develops mild intestinal inflammation in acute infection with Toxoplasma gondii
Source: PLoS One. 2018 Jan 11;13(1):e0190155. doi: 10.1371/journal.pone.0190155 (PMC5764246; doi:10.1371/journal.pone.0190155)

# Experimental Design

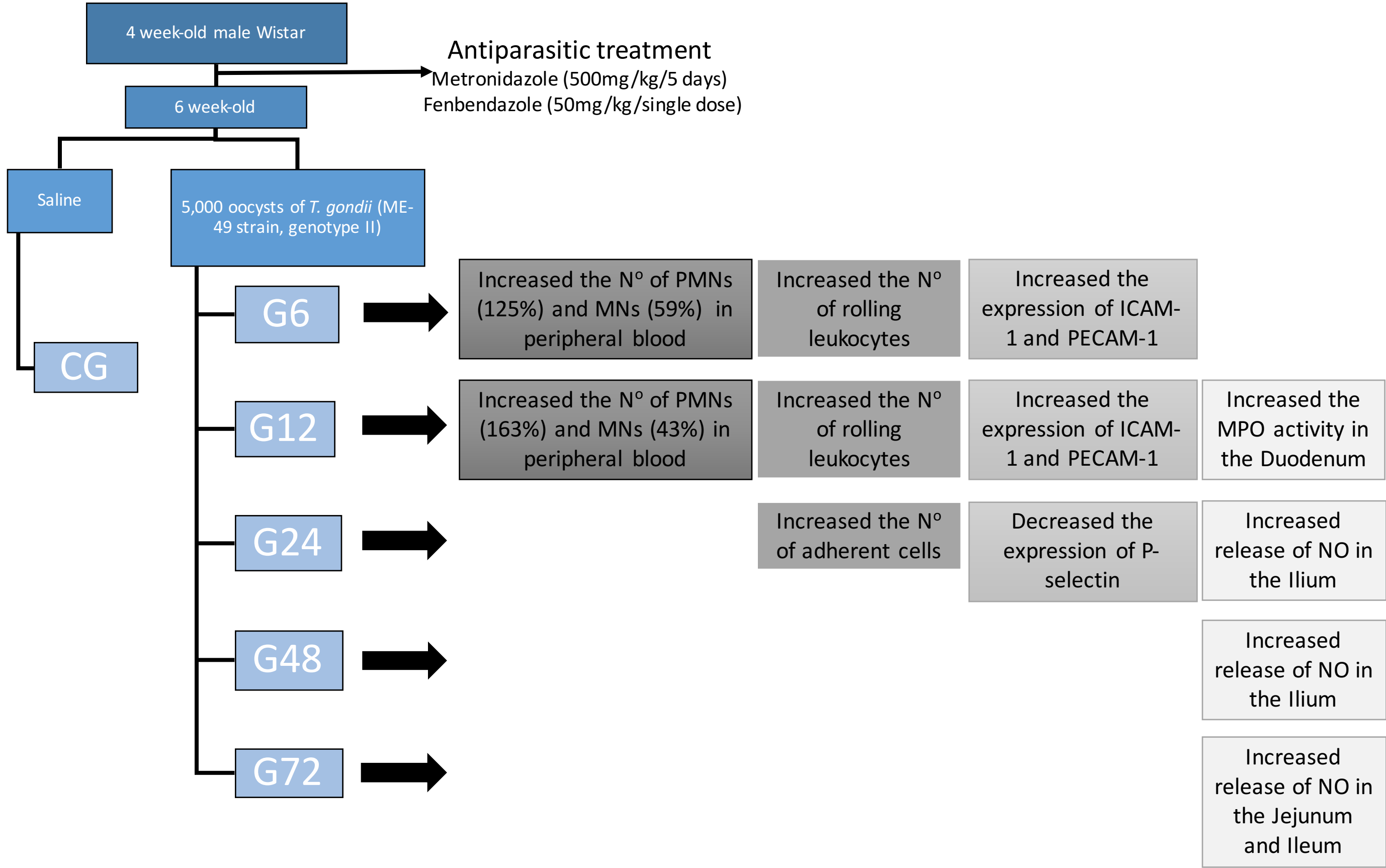

Supplement: S1 Fig — Schematic model exemplifying the experimental model used. (PDF) [file pone.0190155.s001.pdf]
