# Supplementary material for: Immunocompetent host develops mild intestinal inflammation in acute infection with Toxoplasma gondii
Source: PLoS One. 2018 Jan 11;13(1):e0190155. doi: 10.1371/journal.pone.0190155 (PMC5764246; doi:10.1371/journal.pone.0190155)
Supplement: S2 Fig — Schematic illustrating the step-wise protocol of the mesenteric microcirculation in situ in order to analyse the leukocyte endothelium interaction. (PDF) [file pone.0190155.s002.pdf]

Intravital microscopy

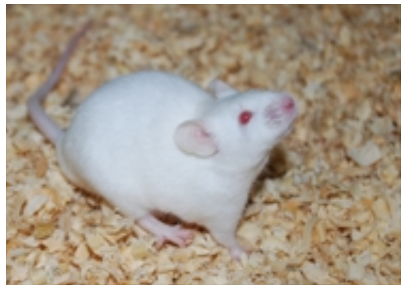

Male Wistar rats
(140-220g)

Anesthesia
intramuscular route
Xylasine (10 mg.kg^{-1})
Ketamine (50 mg.kg^{-1})

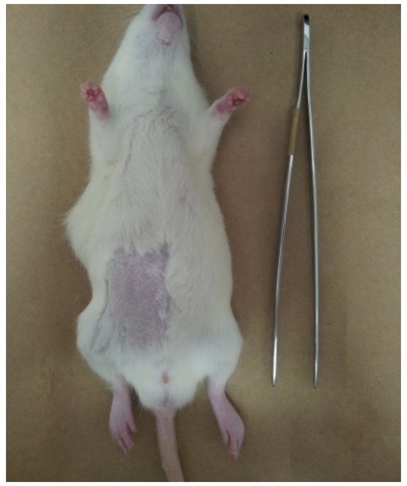

Trichotomy

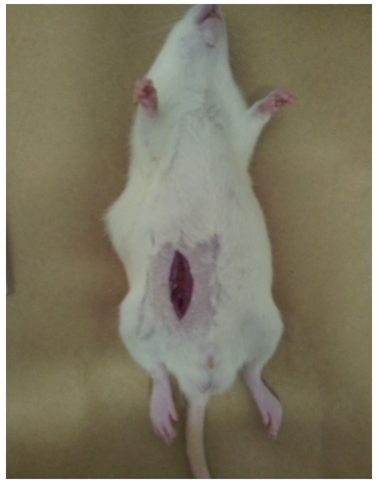

Abdominal incision

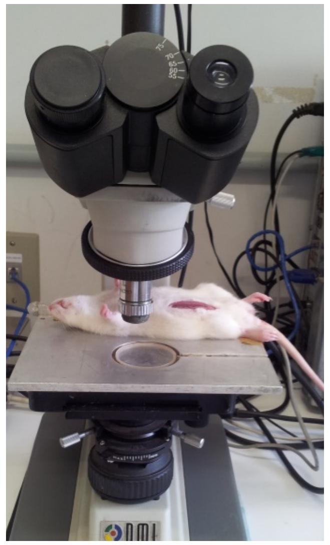

Adaptation on heated plate (37°C) for 5 min

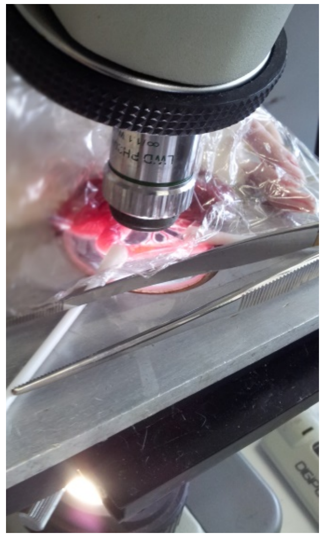

Ileal mesentery exteriorized. The mesenteric exposed for observation of the microcirculation vessels. The preparation was kept moist and warm by irrigation with Ringer-Locke solution (37 ^{o} C, pH 7.2 - 7.4)

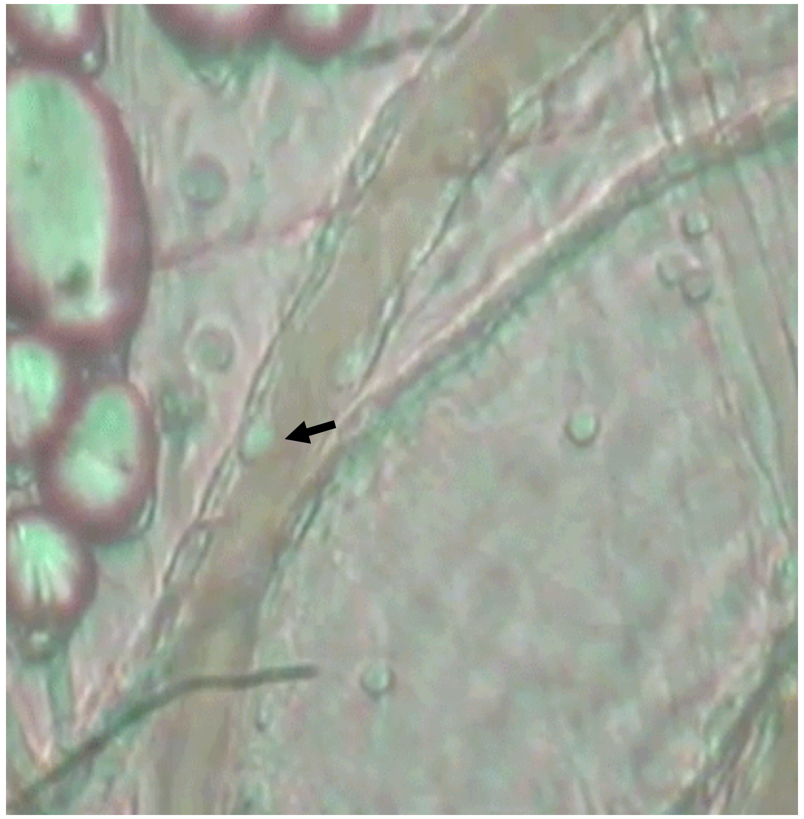

Visualization and analysis of leukocytes. Post-capillary venules with diameters ranging from 18-25 µm. Arrow - leukocyte in rolling.
