# Supplementary material for: Immunocompetent host develops mild intestinal inflammation in acute infection with Toxoplasma gondii
Source: PLoS One. 2018 Jan 11;13(1):e0190155. doi: 10.1371/journal.pone.0190155 (PMC5764246; doi:10.1371/journal.pone.0190155)
Supplement: S1 Table — Total and differential leukocyte count (Cells mm-3) in rat peripheral blood. The rats of the infected groups received orally 5,000 sporulated oocysts of T. gondii (ME-49 strain, genotype II) that were resuspended in 1 mL sterile saline for 6 hours (G6), 12 hours (G12), 24 hours (G24), 48 hours (G48) and 72 hours (G72). The control group (CG) has received 1 mL sterile saline. (PDF) [file pone.0190155.s003.pdf]

Table 1 Total and differential leukocyte count in rat peripheral blood (Cells mm^{-3})

		Groups	N	Average	Standard error
Total leukocyte count		GC	9	7,517	494
		G6	9	12,930	933
		G12	9	12,544	1,495
		G24	8	10,331	390
		G48	10	8,025	482
		G72	9	9,789	538
Differential leukocyte count	Polymorphonuclear	GC	9	1,477	269
		G6	9	3,331	301
		G12	9	3,880	869
		G24	8	2,282	258
		G48	10	1,428	133
		G72	9	2,692	329
	Mononuclear	GC	9	6,040	358
		G6	9	9,597	723
		G12	9	8,665	829
		G24	8	8,049	442
		G48	10	6,597	424
		G72	9	7,097	347
